# Stage-Dependent Succession of Bacterial Communities in the South China Sea Stony Coral *Goniopora* sp. During Bleaching

**DOI:** 10.3390/microorganisms14040833

**Published:** 2026-04-07

**Authors:** Li Mo, Liyu Huang, Xinye Chen, Jiaojiao Zhang, Jiaxin Liu, Jiening Zou, Xiande Huang, Xiaoyong Zhang

**Affiliations:** 1 University Joint Laboratory of Guangdong Province, Hong Kong and Macao Region on Marine Bioresource Conservation and Exploitation, College of Marine Sciences, South China Agricultural University, Guangzhou 510642, China; molly5792@163.com (L.M.); 15889168350@163.com (L.H.); xinyeeast@stu.scau.edu.cn (X.C.); 18617375175@163.com (J.L.); 13421958332@163.com (J.Z.); 2 Biological Physics Laboratory, School of Physics and Astronomy, University of Manchester, Schuster Building, Manchester M13 9PL, UK; jiaojiao.zhang@manchester.ac.uk; 3 College of Food and Health, Zhejiang A & F University, Hangzhou 311300, China

**Keywords:** coral bleaching, *Goniopora* sp., microbial succession, 16S rRNA, coral-associated bacteria

## Abstract

Although coral bleaching–associated microbial changes have been widely studied, bacterial succession during bleaching, particularly in partly bleached corals, remains poorly understood. Here, we investigated bacterial community dynamics in healthy, partly bleached, and bleached *Goniopora* sp. collected from the Sanya Coral Reef Conservation District, South China Sea. A total of 599,003 valid sequences were obtained and clustered into 5094 operational taxonomic units (OTUs). These OTUs spanned 45 bacterial phyla and were identified by 16S rRNA gene sequencing, revealing a highly diverse bacterial community associated with *Goniopora* sp. Alpha diversity differed significantly among health statuses, with partly bleached *Goniopora* sp. (PBG) exhibiting the highest bacterial diversity (Shannon index: 6.25 ± 0.11), followed by bleached *Goniopora* sp. (BG) (5.49 ± 0.18) and healthy *Goniopora* sp. (HG) (3.04 ± 0.17). Beta diversity analyses showed clear separation of microbial community structures among HG, PBG, and BG. Successional analyses revealed a progressive decline in putatively beneficial bacterial taxa, including the phylum Pseudomonadota and the genus *Cohaesibacter* with increasing bleaching severity, whereas the relative abundance of opportunistic or stress-associated bacteria, such as *Blastopirellula*, *Mycobacterium*, and some unclassified taxa, increased. Notably, many bacterial taxa, including Acidobacteriota, *Woeseia* and *Ruegeria*, displayed non-linear abundance patterns, with pronounced shifts during the partly bleached stage. These findings highlight substantial microbial restructuring during coral bleaching and underscore the importance of the partly bleached status as a transitional phase in coral-associated bacterial succession.

## 1. Introduction

Coral reefs are often described as the “rainforests of the sea” due to their exceptionally high biodiversity and ecological importance. Although they occupy less than 1% of the ocean floor, coral reef ecosystems support over a quarter of all known marine species, including sponges, mollusks, and a diverse array of reef-associated fishes [1]. Structurally, coral reef ecosystems are composed of reef-building coral skeletons, associated reef organisms, complex biological communities, and their surrounding physical environment. In addition to their ecological value, corals and reef-associated algae support fisheries, provide pharmaceutical resources, and underpin marine ecotourism, thereby providing important ecosystem services that sustain coastal economies and promote sustainable marine development [2,3].

Despite their importance, coral reef ecosystems are increasingly threatened by environmental stressors, with large-scale coral bleaching having emerged as one of the most severe and widespread disturbances [4,5]. Over recent decades, coral bleaching events have been reported from more than 50 countries worldwide. During the 2015–2017 global bleaching period, more than 30% of coral cover was lost across major reef systems [6]. In 2016, reefs in the western Indian Ocean experienced extensive bleaching and mortality in response to elevated sea surface temperatures [4,7]. Recurrent bleaching events have subsequently been documented in the Great Barrier Reef and in several regions of the South China Sea, including the Beibu Gulf, Xisha Islands, and Nansha Islands [8]. Earlier mass bleaching events, such as those recorded in the Seychelles in 1998, further demonstrate that coral bleaching is neither spatially nor temporally isolated [9]. Large-scale bleaching events often result in substantial coral mortality, leading to sharp declines in live coral cover and species diversity [4,5,10]. This loss reduces habitat complexity, negatively impacting associated reef organisms and fisheries productivity, and ultimately compromises the stability and functioning of marine ecosystems [8].

The mechanisms underlying coral bleaching have been widely discussed. Some studies have suggested that bleaching-prone reefs exhibit increased sponge abundance, implying potential competitive interactions between sponges and corals [11]. Other research has demonstrated that elevated sea surface temperatures and increased solar radiation disrupt the symbiosis between corals and their endosymbiotic dinoflagellates, resulting in bleaching and coral mortality [5,12,13,14]. Mounting evidence indicates that changes in coral-associated microbial communities play a pivotal role in bleaching processes [15,16]. Alterations in bacterial community structure and diversity within coral tissues and surface mucus layers can destabilize the coral holobiont, culminating in microbial dysbiosis and increased bleaching susceptibility [13]. In particular, several studies have linked coral bleaching to pathogenic bacteria, especially members of the genus *Vibrio* [17,18]. Elevated temperatures may enhance bacterial virulence while simultaneously reducing the abundance or activity of beneficial microbial taxa; this, in turn, drives declines in symbiotic zooxanthellae and accelerates bleaching progression [13]. Conversely, recent studies have suggested that corals harboring higher bacterial diversity, richness, and community evenness may exhibit greater resistance to bleaching, whereas shifts in community composition and antagonistic capacity can influence coral health during stress events [15,19]. Nevertheless, much of the research to date has focused primarily on comparisons between healthy and fully bleached corals [20], whereas intermediate bleaching stages and the associated community-level structural changes remain comparatively understudied.

This study was designed to elucidate stage-specific bacterial succession during coral bleaching, with an emphasis on the ecological significance of the partly bleached status in microbial community restructuring. Focusing on the scleractinian coral *Goniopora* sp., we analyzed samples representing three health statuses: healthy, partly bleached, and bleached. High-throughput 16S rRNA gene sequencing was employed to systematically compare bacterial diversity, community composition, and successional patterns across bleaching stages. Furthermore, shifts in bacterial taxa at the phylum and genus levels were examined to characterize dynamic changes in putatively beneficial bacteria and opportunistic or stress-associated taxa during the bleaching process, thereby providing comprehensive microbiological evidence for understanding coral–microbe interactions under environmental stress.

## 2. Materials and Methods

### 2.1. Goniopora sp. Sample Collection

The coral *Goniopora* sp., a common reef-building scleractinian coral widely distributed in the Indo-Pacific region, is frequently observed on shallow reefs in the Sanya Coral Reef Conservation District, the South China [21]. *Goniopora* sp. is locally abundant and has been observed to exhibit visible bleaching under periods of elevated sea surface temperature [22], making it suitable for investigating bacterial community dynamics associated with coral bleaching.

Three biological replicates (*n* = 3) for each group—healthy *Goniopora* sp. (HG), partly bleached *Goniopora* sp. (PBG) and bleached *Goniopora* sp. (BG)—were collected from the Sanya Coral Reef Conservation District (18°10′ N, 109°25′ E), South China Sea, in October 2021 [23]. Coral colonies were identified in situ based on macromorphological characteristics, including large fleshy polyps with extended tentacles and a massive, porous calcareous skeleton typical of the genus *Goniopora* sp. Immediately after collection, samples were placed into sterile, labeled sampling bags under aseptic conditions, stored in a low-temperature container, and transported to the laboratory as soon as possible for subsequent analyses.

### 2.2. DNA Extraction

Total genomic DNA was extracted from preserved *Goniopora* sp. fragments following sequential rinsing with 75% ethanol and sterile seawater to remove loosely attached microorganisms and surface mucus [23]. DNA extraction was performed using the E.Z.N.A.^®^ Soil DNA Kit (Omega Bio-tek, Norcross, GA, USA) according to the manufacturer’s instructions, with minor modifications to improve coral tissue homogenization. Specifically, coral fragments were aseptically homogenized in sterile lysis buffer using a sterile mortar and pestle or bead-beating disruptor tubes to ensure efficient disruption of microbial cells embedded within the coral matrix. DNA concentration and purity were assessed using a NanoDrop 2000 spectrophotometer (Thermo Fisher Scientific, Wilmington, DE, USA), with acceptable purity defined as A260/A280 ratios between 1.8 and 2.0 and A260/A230 values > 1.8. Extracted DNA was stored at −20 °C until further analysis [24].

### 2.3. 16S rRNA Gene Amplification

The hypervariable V4 region of the bacterial 16S rRNA gene was amplified using the universal primers 515F (5′-GTGYCAGCMGCCGCGGTAA-3′) and 907R (5′-CCGTCAATTCMTTTRAGTTT-3′), which provide broad coverage of bacterial taxa commonly associated with marine and coral microbiomes [25]. PCR amplification was carried out in 25 μL reaction volumes containing 1× PCR buffer, 2 mM MgCl_2_, 0.2 mM of each dNTP, 0.4 μM of each primer, 1 U Taq DNA polymerase (or a high-fidelity equivalent), and 10–50 ng of template DNA. The thermal cycling program consisted of an initial denaturation at 94 °C for 3 min, followed by 30 cycles of denaturation at 94 °C for 30 s, annealing at 52 °C for 30 s, and extension at 72 °C for 45 s, with a final extension at 72 °C for 10 min. No-template negative controls were included in each PCR run to monitor potential contamination [23].

### 2.4. Amplicon Purification and High-Throughput Sequencing

PCR products (~400 bp) were confirmed by electrophoresis on 1.5% agarose gels and subsequently purified using the GeneJET Gel Extraction Kit (Thermo Scientific, Vantaa, Finland) following the manufacturer’s protocol. Purified amplicons were quantified using a fluorometric method (e.g., Qubit dsDNA HS Assay Kit, Invitrogen, Carlsbad, CA, USA), and equimolar amounts of each sample were pooled to construct sequencing libraries. The libraries were sequenced on an Illumina MiSeq platform (Illumina, San Diego, CA, USA) using paired-end reads (2 × 300 bp), which are suitable for V4-region amplicon sequencing [14,25]. The raw sequencing data from unbleached, partly bleached, and bleached colonies of the stony coral *Goniopora* sp. have been deposited in the NCBI Sequence Read Archive (SRA) under BioProject accession PRJNA1420048.

### 2.5. Sequence Processing and Microbial Community Analysis

Raw sequencing reads were processed and analyzed using the QIIME2-2023.2 software package [26]. Illumina raw reads were demultiplexed based on barcode sequences, allowing up to one nucleotide mismatch. Barcode sequences, primers, and low-quality reads were removed to obtain high-quality paired-end reads. Paired-end reads were merged using FLASH 1.2.11. Operational taxonomic units (OTUs) were clustered at 97% sequence similarity using UPARSE (v7.0.1001) [25]. Taxonomic classification was performed with the RDP classifier against the Silva 16S rRNA gene database (Version 138). Prior to downstream analyses, unclassified OTUs, chloroplast-derived sequences, and singleton OTUs were excluded. Alpha diversity indices, including Chao1 and Shannon indices, were calculated using Mothur 1.21.1, and rarefaction curves were generated based on Chao1, observed species richness, and Shannon indices [14]. Microbial sequence numbers, OTU richness, and Shannon indices for *Goniopora* sp. samples were expressed as mean values [23,26].

### 2.6. Beta Diversity and Statistical Analysis

Beta diversity analysis was conducted based on Bray–Curtis distances and visualized using principal component analysis (PCA). Differences in microbial community composition among *Goniopora* samples with different health statuses were assessed using analysis of similarities (ANOSIM), as commonly applied in microbial community ecology studies [26].

## 3. Results

### 3.1. Illumina Sequencing and Sequence Analysis

After rigorous quality filtering, denoising, and chimera detection, a total of 599,003 valid 16S rRNA V4 sequences were retained for downstream analysis. These high-quality sequences were subsequently clustered into 5094 OTUs based on 97% similarity. The numbers of bacterial sequences recovered from HG, PBG and BG were 58,457 ± 2799, 55,418 ± 5087 and 50,617 ± 1774, respectively. The corresponding OTU counts were 735.33 ± 29.97, 2835.67 ± 187.26 and 1420.67 ± 306.36, respectively (Table 1). Rarefaction curves were constructed based on OTU counts and sequencing depth. The asymptotes of sequence tracks of samples under different health statuses were nearly parallel, indicating that the number of bacterial sequences obtained in the study was close to saturation. Furthermore, Shannon’s index calculated for HG, PBG and BG samples was 3.04 ± 0.17, 6.25 ± 0.11 and 5.49 ± 0.18, respectively. These results collectively indicate that corals across different health statuses harbor diverse bacterial communities.

### 3.2. Diversity and Composition of Bacterial Communities in Goniopora sp.

**Figure 1:**
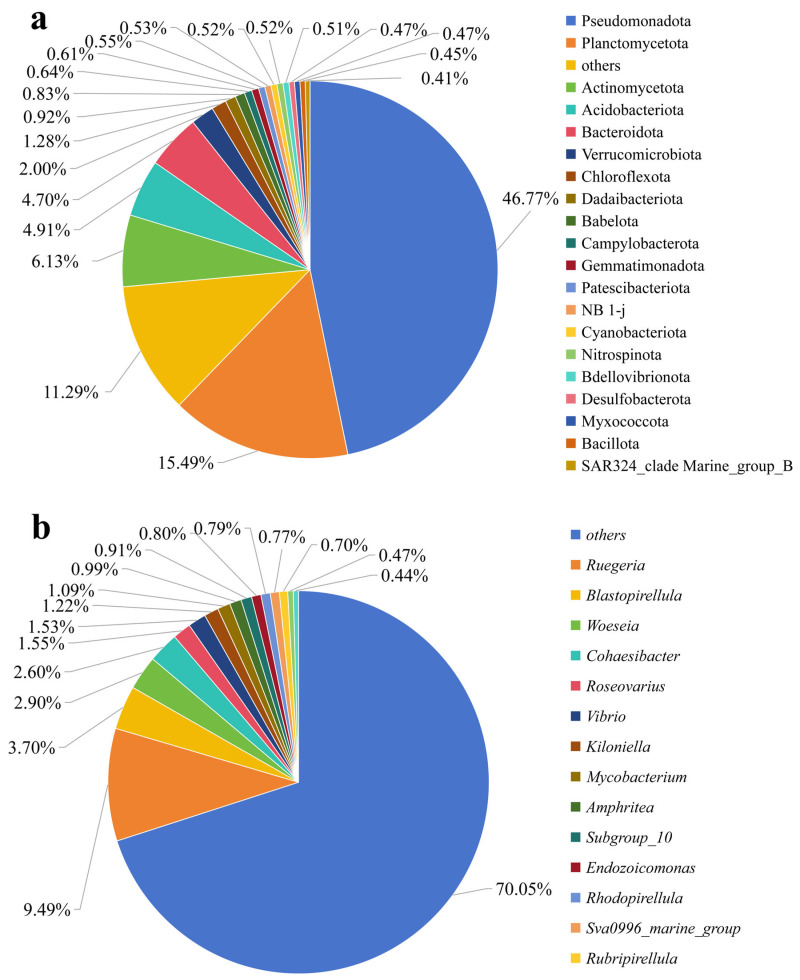
Relative abundance of bacterial communities associated with *Goniopora* sp. based on 16S rRNA gene sequencing. (**a**) Bacterial community composition at the phylum level. (**b**) Relative abundance of classified bacterial genera. Relative abundances are expressed as percentages of total valid sequences, with low-abundance taxa (less than 0.4%) grouped as “others”.

A total of 5094 bacterial OTUs from 45 phyla were detected across all coral samples. *Pseudomonadota* was the most abundant phylum, accounting for 46.77% of the total bacterial communities (with a mean relative abundance across all samples), followed by *Planctomycetota* (15.49%), *Actinomycetota* (6.13%), *Acidobacteriota* (4.91%), *Bacteroidota* (4.70%), *Verrucomicrobiota* (2.00%), and *Chloroflexota* (1.28%) (Figure 1a). In addition, other phyla with lower abundance were detected, including *Dadaibacteriota* (0.92%), *Babelota* (0.83%), *Campylobacterota* (0.64%), *Gemmatimonadota* (0.61%), *Patescibacteriota* (0.55%), NB 1-j (0.53%), *Cyanobacteriota* (0.52%), *Nitrospinota* (0.52%), *BdelloVibrionota* (0.51%), *Desulfobacterota* (0.47%), *Myxococcota* (0.47%), *Bacillota* (0.45%), SAR324_cladeMarine_group_B (0.41%).

At the genus level, the identified genera were dominated by *Ruegeria*, which accounted for 9.49% of the total communities, *Blastopirellula* (3.70%), and Woeseia (2.90%) (Figure 1b). *Cohaesibacter* (2.60%) and *Roseovarius* (1.55%) also occupied notable proportions. Other identified genera included *Vibrio* (1.53%), *Kiloniella* (1.22%), and *Mycobacterium* (1.09%). Genera with relative abundances below 1% included *Amphrite* (0.99%), *Subgroup_10* (0.91%), *Endozoicomonas* (0.80%), *Rhodopirellula* (0.79%), *Sva0996_marine_group* (0.77%), *Rubripirellula* (0.70%), *Halarcobacter* (0.47%), and *Candidatus_Berkellia* (0.44%). Although these taxa had relatively low abundances, they may have specific ecological functions within the coral bacterial communities. Surprisingly, a large number of previously unclassified taxa were also found in these coral samples, collectively accounting for at least 35.99%. This high prevalence of unclassified sequences underscores the immense and still largely uncharacterized microbial “dark matter” within the *Goniopora* sp. holobiont. The presence of such a vast reservoir of unclassified bacteria suggests that many key microbial players, which may be crucial for host physiological adaptation and resilience during thermal stress, remain to be formally identified. Future studies employing metagenomic assembly or cultivation-dependent approaches are warranted to further resolve the taxonomic identities and functional roles of these unassigned microbial members.

### 3.3. Comparative Analysis of Variability Among Bacterial Communities

**Figure 2:**
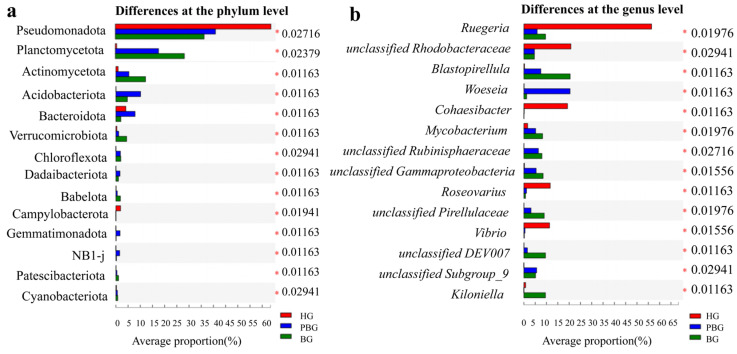
Differentially abundant bacterial taxa associated with *Goniopora* sp. (**a**) Bacterial phyla showing significant differences in relative abundance among groups. (**b**) Bacterial genera showing significant differences in relative abundance among groups. Bars represent the average relative abundance (%) of each taxon. Asterisks (*) indicate statistically significant differences, with the corresponding *p* values shown on the right.

Principal coordinate analysis (PCoA) (R = 0.93, *p* = 0.001) and non-metric multidimensional scaling (NMDS) (R = 1.00, *p* = 0.001) revealed distinct clustering of bacterial communities among the three health statuses. This indicates a strong correlation between coral health status and bacterial community composition. At the phylum level, significant differences in relative abundance were observed for multiple taxa (Figure 2a). Specifically, *Actinomycetota*, *Acidobacteriota*, *Bacteroidota*, *Chloroflexota*, *Dadaibacteriota*, *Babelota*, *Gemmatimonadota*, NB1-j, *Patescibacteriota*, and *Cyanobacteriota* showed significant differences across health statuses. Additionally, *Planctomycetota* and *Pseudomonadota* also showed significant differences. At the genus level (Figure 2b), significant differences were observed in *Blastopirellula*, *Woeseia*, *Cohaesibacter*, *Roseovarius*, and *Kiloniella*, with *Vibrio* showing relatively pronounced differences as well. Notable variations were also detected in *Ruegeria* and *Mycobacterium*. Among unclassified genera, *unclassified DEV007* exhibited the most striking difference, followed by *unclassified Gammaproteobacteria*, which also showed a clear distributional distinction. Additionally, *unclassified Rubinisphaeracea*, *unclassified Subgroup_9*, and *unclassified Rhodobacteraceae* displayed significant differences.

### 3.4. Succession of Bacteria Community in the Process of Goniopora sp. Bleaching

**Figure 3:**
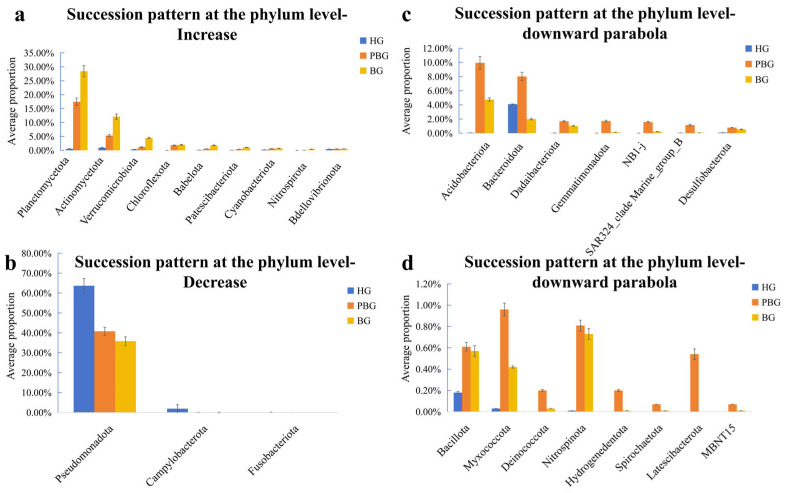
Successional patterns of bacterial phyla during coral bleaching. (**a**) Phyla showing increasing relative abundance from HG to PBG and BG. (**b**) Phyla showing decreasing relative abundance along the bleaching gradient. (**c**,**d**) Phyla exhibiting parabolic succession patterns across coral bleaching stages. Bars indicate mean relative abundance (%), and error bars represent standard deviation.

After a detailed analysis of the bacterial community’s structure during coral bleaching, three distinct patterns of succession were identified across three stages: healthy *Goniopora* sp. (HG), partly bleached *Goniopora* sp. (PBG), and bleached *Goniopora* sp. (BG). Initially, the bacterial abundance exhibited a consistent increase across the bleaching gradient (Figure 3a). For instance, Planctomycetota increased from 0.57% in HG to 17.46% in PBG, and further to 28.44% in BG. Similarly, Actinomycetota rose from 0.94% in HG to 5.32% in PBG, and eventually to 12.13% in BG. Verrucomicrobiota also showed a significant rise, increasing from 0.37% in HG to 1.19% in PBG, and then to 4.46% in BG. Other bacterial groups, such as Chloroflexota, Babelota, Patescibacteriota, Cyanobacteriota, and Nitrospirota, exhibited similar increasing trends. The second pattern was a consistent decrease across the bleaching gradient (Figure 3b). For example, Pseudomonadota decreased from 63.67% in HG to 40.79% in PBG to 35.85% in BG. Interestingly, Campylobacterota and Fusobacteriota, which accounted for 1.92% and 0.08% in HG, respectively, were nearly undetectable in PBG and BG. A third pattern was parabolic (i.e., non-linear) abundance dynamics across the three stages. Specifically, it showed an upward trend from HG to PBG and then a downward trend from PBG to BG (Figure 3c,d). For example, Acidobacteriota increased from 0.05% in HG to 9.94% in PBG and then decreased to 4.75% in BG, while Bacteroidota increased from 4.10% in HG to 8.02% in PBG and then decreased to 1.99% in BG. Other phyla, including Dadaibacteriota, Gemmatimonadota, NB 1-j, SAR324_cladeMarine_group_B, Desulfobacterota, Bacillota, Myxococcota, Deinococcota, and Nitrospinota, exhibited similar parabolic patterns. It is worth noting that Spirochaetota, Latescibacterota, Hydrogenedentota, and MBNT15 were detected exclusively in PBG, with proportions of 0.07%, 0.54%, 0.20%, and 0.07%, respectively.

**Figure 4:**
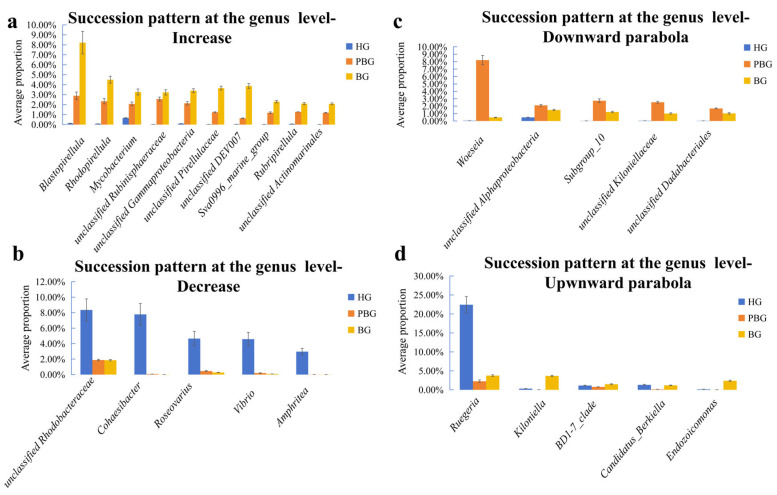
Successional patterns of bacterial genera during coral bleaching. (**a**) Genera showing increasing relative abundance from HG to PBG and BG. (**b**) Genera showing decreasing relative abundance along the bleaching gradient. (**c**) Genera exhibiting downward parabolic succession patterns across coral bleaching stages. (**d**) Genera exhibiting upward parabolic succession patterns across coral bleaching stages. Bars indicate mean relative abundance (%), and error bars represent standard deviation.

At the genus level, the abundance of bacterial genera showed consistent increasing trends across the bleaching gradient (Figure 4a). For example, *Blastopirellula* increased from 0.11% in HG to 2.89% in PBG and further to 8.21% in BG. Similarly, *Mycobacterium* rose from 0.66% in HG to 2.07% in PBG and eventually to 3.27% in BG. *Unclassified Gammaproteobacteria* also showed a significant rise, increasing from 0.09% in HG to 2.13% in PBG, and then to 3.40% in BG. Other genera, such as *Rhodopirellula*, *unclassified Rubinisphaeraceae*, *unclassified Pirellulaceae*, *unclassified DEV007*, *Sva0996_marine_group*, *Rubripirellula*, and *unclassified Actinomarinales*, exhibited similar increasing trends. Several genera exhibited consistent decreasing trends across the bleaching gradient (Figure 4b). For instance, the most pronounced decline was observed in the genus *Cohaesibacter*, which decreased dramatically from 7.77% in HG to only 0.07% in PBG, and further to 0.02% in BG. Similarly, the genus *Vibrio* also showed a continuous decline from 4.58% in HG to 0.20% in PBG and then to 0.08% in BG. Other genera that exhibited a similar decreasing trend include *Roseovarius*, *Amphritea*, and *unclassified Rhodobacteraceae*. Several genera exhibited non-linear (parabolic) abundance patterns, which could be further categorized into two distinct subtypes. The first pattern was characterized by an increase from HG to PBG followed by a decrease from PBG to BG (Figure 4c). For example, *Woeseia* increased from 0.05% in HG to 8.21% in PBG but then decreased to 0.49% in BG. Similarly, *Subgroup_10* increased from 0.02% in HG to 2.74% in PBG and then decreased slightly to 1.22% in BG. The same trend was observed for *unclassified Dadabacteriales*, *unclassified Kiloniellaceae,* and *unclassified Alphaproteobacteria*. The second pattern was characterized by a decrease from HG to PBG, followed by an increase from PBG to BG (Figure 4d). For example, *Ruegeria* decreased dramatically from 22.44% in HG to 2.27% in PBG and then increased slightly to 3.77% in BG. Similarly, *Kiloniella* decreased from 0.31% in HG to 0.00% in PBG but then increased to 3.67% in BG. The same trend was observed for *Candidatus_Berkiella*, *BD1-7_clade*, and *Endozoicomonas*.

## 4. Discussion

Corals harbor diverse microbial communities that play essential roles in host health and resilience, making them a long-standing focus of marine ecological research [16]. To characterize the composition and diversity of microbial communities associated with *Goniopora* sp., this study employed high-throughput 16S rRNA gene sequencing. A total of 45 distinct bacterial phyla were identified from *Goniopora* sp. samples (Figure 1a), revealing a surprisingly rich and diverse microbial community that substantially expands our understanding of coral-associated microbial diversity. In addition to known taxa, at least 35.99% of the bacteria associated with *Goniopora* sp. remained unidentified, including *unclassified Rhodobacteraceae* and *unclassified Rubisphaeraceae*. These unclassified taxa likely represent novel microbial lineages, suggesting that coral ecosystems harbor a substantial number of undiscovered species.

Although the majority of the bacterial phyla recovered from the coral had been previously reported in coral-associated microbial studies, this study is the first to report the presence of 15 bacterial phyla in corals, including Babelota, NB1-j, Myxococcota, Latescibacterota, Deinococcota, Hydrogenedentota, Sumeraeota, MBNT15, Marinimicrobia_SAR406_clade, Elusimicrobiota, Fibrobacterota, RCP2-54, Calditrichota, PAUC34, and WS2. In marine sediments, certain bacterial phyla have been frequently reported. For instance, NB1-j, Myxococcota [27], Calditrichota and Fibrobacterota [28] have all been documented. Among these, Myxococcota has been characterized as a widely distributed micro-predator in global sediments [29], whereas Deinococcota has been identified as a reservoir of antibiotic resistance genes (ARGs) in marine sediments [30]. Additionally, some of these phyla have been detected in the guts of marine organisms. For example, Dependentiae has been reported in the gut of carp and large yellow croaker [31,32], Fibrobacterota in Symphysodon aequifasciatus [33], and Marinimicrobia_SAR406_clade in the swimming crab Portunus trituberculatus [34]. Other phyla, such as Latescibacterota, are widely distributed in estuarine and marine ecosystems, with their abundance increasing with water depth [35]. In contrast, phyla such as Hydrogenedentota, Sumeraeota, MBNT15, RCP2-54, and WS2, while more commonly observed in other ecosystems, have rarely been reported in marine environments. These findings extend our understanding of coral-associated microbial diversity.

This study revealed that the relative abundance of the same bacterial taxa varied significantly across the three health statuses. This suggests that factors such as the decline of beneficial bacteria or the proliferation of opportunistic pathogens may contribute to bleaching in *Goniopora* sp. [36]. At the phylum level, the relative abundance of Pseudomonadota showed a consistent decreasing trend across HG, PBG, and BG (Figure 3b). Most Pseudomonadota are beneficial for *Goniopora* sp. They are known to participate in nutrient supply, metabolic regulation, and antioxidant capacity in corals, and are often enriched in bacterial communities associated with stress resistance [37,38]. A similar decreasing trend was observed at the genus level for *Cohaesibacter*, *Roseovarius*, and *Vibrio* (Figure 4b). Among these, *Cohaesibacter* has been positively correlated with immune gene expression and beneficial immune function in fish [39,40]. *Roseovarius* possesses genomic traits that enhance bacterial colonization and association with coral hosts [41] and exhibits mild anti-algal and antibacterial activity [42]. These bacteria are expected to play active protective roles in corals under normal conditions. However, our results showed the opposite trend. This decline may reflect the degradation of the microbial communities during the coral bleaching, weakening the coral’s microbial defense mechanisms and reducing antioxidant capacity, leading to increased reactive oxygen species (ROS) levels, which can lead to increased reactive oxygen species (ROS) levels—a process linked to oxidative stress and bleaching progression under thermal stress [41]. Concurrently, this microbial shift may compromise coral stress resistance, increasing susceptibility to environmental stress and thereby exacerbating bleaching.

When coral bleaching occurs, the loss of symbiotic dinoflagellates disrupts host metabolic balance and immune regulation [19], which may facilitate the proliferation of opportunistic or potentially pathogenic microorganisms, ultimately leading to pronounced shifts in the coral-associated microbial community structure [43,44]. At the phylum level, Verrucomicrobiota, Babelota, Patescibacteriota, and Cyanobacteriota exhibited a consistent increase in relative abundance from HG to PBG and BG (Figure 3a), suggesting that these taxa may be progressively favored as coral–microbe homeostasis deteriorates during bleaching [43]. Verrucomicrobiota are widely distributed in marine ecosystems [45] and are often associated with the degradation of complex polysaccharides and organic matter [46]. Their enrichment in bleached corals may therefore reflect increased availability of host-derived organic substrates following tissue damage and mucus alteration, rather than a stable mutualistic association. Babelota (TM6) are characterized by highly reduced genomes and a strong dependence on host-associated lifestyles, and the first isolated representative of this phylum was shown to possess genomic features highly specialized for infection [47,48]. The increasing abundance of Babelota observed in this study suggests that destabilized host conditions during bleaching may promote the proliferation of bacteria with parasitic or epibiotic tendencies, potentially imposing additional stress on coral hosts. Patescibacteriota, also referred to as the Candidate Phyla Radiation (CPR) [49], are broadly distributed in marine water columns, sediments, and host-associated microbiomes, and are characterized by small genomes, limited biosynthetic capacities, and strong metabolic dependence on surrounding microorganisms [50]. Genome-resolved studies in marine sediments have shown that Patescibacteriota are often enriched in disturbed or simplified microbial ecosystems, where metabolic interdependencies among microorganisms become more pronounced [51]. The consistent increase in Patescibacteriota abundance from HG to BG observed in this study may therefore indicate progressive microbial community simplification and functional instability during coral bleaching, rather than a direct beneficial role for the coral host. In addition, Cyanobacteriota exhibited a pronounced increase in bleached corals, highlighting their potential contribution to bleaching-associated microbial dysbiosis. Certain Cyanobacteriotal taxa are known to produce a wide range of bioactive and toxic secondary metabolites [52] and can negatively impact surrounding organisms and environments through toxin release, oxygen depletion, and deterioration of water quality [53]. The accumulation of Cyanobacteriota during bleaching may further exacerbate host stress and hinder recovery, reinforcing a negative feedback loop between microbial imbalance and coral degradation. Among the significantly different genera, *Blastopirellula* and *Mycobacterium* showed increasing trends during the coral bleaching (Figure 4a). Some species of *Mycobacterium* are pathogenic. For example, *Mycobacterium* marinum is a well-known pathogen that infects freshwater and marine fish, causing necrotic granulomas and associated morbidity and mortality [54]. Corals, which serve as habitats for various fish species, exhibit weakened defense mechanisms during bleaching [19,55]. The increase in *Mycobacterium* abundance raises the likelihood of opportunistic pathogen invasion, further compromising coral health and exacerbating bleaching [43,44]. Meanwhile, the enrichment of *Mycobacterium* may disrupt coral metabolic processes by competing for nutrients or releasing harmful metabolic by-products, thereby affecting normal physiological functions of the host. Bleached corals rely on heterotrophic uptake and microbial functional compensation to facilitate recovery [56]. However, increased *Mycobacterium* abundance may interfere with this recovery process by competing for essential nutrients [57] or producing toxic metabolites [58], ultimately reducing coral resilience and recovery capacity. Overall, the consistent enrichment of Verrucomicrobiota, Babelota, Patescibacteriota, and Cyanobacteriota across bleaching stages suggests a shift from a relatively stable, host-associated microbial community toward one dominated by opportunistic and stress-associated taxa, reflecting the progressive breakdown of coral–microbe homeostasis during bleaching.

In this study, we observed that the abundance of certain bacterial taxa in bleached corals consistently increased. These taxa may indirectly reflect coral health status and bleaching susceptibility through their interactions with other microbial taxa [59]. Coral bleaching also significantly alters microbial metabolic functions, particularly those related to carbon cycling and polysaccharide degradation [60]. *Blastopirellula* may participate in nutrient cycling and metabolic processes within the coral holobiont [61] (Figure 4a). During bleaching, some microorganisms release organic compounds that promote the proliferation of opportunistic bacteria [43,44]; *Blastopirellula* may influence the progression of coral bleaching by modulating these microbial interactions [60,61]. However, our study did not provide detailed taxonomic classification of *unclassified Rubinisphaeraceae*, *unclassified Gammaproteobacteria*, *unclassified Pirellulaceae*, *unclassified DEV007* and *unclassified Subgroup_9* (Figure 3b). Therefore, it remains challenging to assess the specific roles of these taxa in coral bleaching processes.

A key innovation of this study, compared with previous coral microbiome research, is the inclusion of partly bleached coral samples, which enabled the identification of transitional microbial shifts during the bleaching process [20]. At the phylum level, Acidobacteriota, Bacteroidota, Dadaibacteriota, Gemmatimonadota, and NB1-j exhibited a consistent pattern, with relative abundances increasing from HG to PBG, followed by a marked decrease in BG (Figure 3c,d). This trend suggests that these bacterial phyla may be actively recruited or enriched during early environmental stress but decline as bleaching progresses and host physiological regulation becomes impaired [62].

Environmental stressors such as elevated temperature, high light intensity, salinity fluctuations, inorganic nutrient imbalance, trace metal exposure, and altered nitrogen-to-phosphorus ratios can induce excessive accumulation of reactive oxygen species (ROS) and reactive nitrogen species (RNS) in coral tissues, thereby disrupting host–symbiont homeostasis [26,41,63]. Several bacterial phyla enriched during the partly bleached stage possess metabolic capabilities potentially beneficial for stress mitigation, including detoxification, nutrient recycling, and oxidative stress regulation [20,41,61]. Gemmatimonadota are known to participate in the degradation of complex organic carbon, denitrification, sulfate reduction, and sulfur oxidation, which may help alleviate oxidative and chemical stress in corals [64,65]. Dadaibacteriota are frequently detected in polluted environments and exhibit resistance to heavy metals [66] and aromatic compounds [67], partly through EPS-mediated detoxification and denitrification processes [68]. Similarly, Acidobacteriota and Bacteroidota have been reported as beneficial or stress-tolerant taxa, with functions including heavy metal resistance, nutrient fixation, and pollutant removal [69,70,71,72]. While the specific ecological roles of NB1-j in corals remain to be fully elucidated, its significant enrichment during the partly bleached stage suggests that this taxon may be an active participant in the microbial restructuring associated with environmental stress.

The subsequent decline in the relative abundance of these potentially beneficial bacterial phyla in fully bleached corals may indicate a reduction in microbial-mediated protective functions, which could exacerbate host vulnerability and accelerate bleaching progression [44]. This observation highlights the partly bleached stage as a critical transitional period during which the coral-associated microbiome still retains a certain capacity for stress buffering [15]. At the genus level, distinct functional differentiation was observed among key bacterial taxa. *Ruegeria*, a well-documented potential probiotic associated with healthy corals [73,74], possesses genomic traits that enhance host colonization and symbiotic stability [41]. In particular, *Ruegeria* profundi has been shown to inhibit pathogenic *Vibrio* species and confer protection against coral bleaching [73]. *Kiloniella*, a member of the Rhodobacteraceae, is also commonly associated with healthy coral microbiomes and is thought to contribute to nutrient cycling and host resilience. The observed decline in *Ruegeria* and *Kiloniella* abundance during the partly bleached stage (Figure 4d) suggests a weakening of these protective microbial functions, potentially reducing the coral’s ability to cope with escalating environmental stress. In contrast, *Woeseia* showed an opposite pattern and was enriched under bleaching conditions (Figure 4c). *Woeseia* has been widely reported as a stress-associated or opportunistic taxon, frequently detected in disturbed or organically enriched environments, and is involved in enhanced carbon and sulfur cycling [20,75]. Its enrichment during coral bleaching may reflect a shift toward a microbiome structure dominated by taxa adapted to degraded or unstable conditions rather than host-protective symbiosis. Together, these results indicate that coral bleaching is accompanied by a transition from a microbiome dominated by potentially beneficial bacteria to one increasingly shaped by stress-associated taxa, with the partly bleached stage representing a key turning point in microbial community restructuring.

## 5. Conclusions

Systematic comparisons of *Goniopora* sp. across HG, PBG and BG revealed significant shifts in bacterial diversity and distinct community succession patterns during coral bleaching. The results showed that PBG exhibited the highest bacterial richness and diversity, harboring a large number of taxonomically unclassified bacterial groups, which reflected the high heterogeneity and dynamic instability of microbial communities at this stage. Community structure analysis indicated that as bleaching severity increased, certain potentially beneficial bacterial groups (e.g., Pseudomonadota and associated genera) continued to decline, while multiple bacteria associated with environmental stress or opportunistic lifestyles—such as Verrucomicrobiota and Babelota—gradually became enriched. Notably, the partly bleached stage did not represent a simple transition between healthy and fully bleached conditions, but rather constituted a critical succession node characterized by nonlinear changes, reflecting the reorganization process of the coral–microbe system under environmental stress. The increased diversity and complex succession trajectory observed during this stage suggests that microbial instability may have accelerated coral bleaching. This study highlights the critical research value of the intermediate bleaching stage in evaluating coral–microbe interactions under environmental stress. Nevertheless, several limitations of this study should be acknowledged. We acknowledge that the sample size in this study (*n* = 3 per group) may limit the breadth of generalization for some microbial successional patterns, particularly for those exhibiting complex non-linear dynamics. However, the high degree of similarity among biological replicates and the alignment of our results with established coral bleaching models suggest that these findings capture the core microbial transitions. This work provides an essential baseline for *Goniopora* sp., and future research with expanded sampling will be critical to further refine these ecological successional models.

## Figures and Tables

**Table 1:** Alpha diversity indices in *Goniopora* sp. bacterial communities: HG (healthy, *n* = 3), PBG (partly bleached, *n* = 3), BG (fully bleached, *n* = 3).

Samples	Ace	Chao	Shannon	Sobs
HG1	968.41	949.01	2.94	702
HG2	998.33	993.36	2.94	760
HG3	886.97	884.98	3.24	744
Average of HG	951.24 ± 57.63	942.45 ± 54.49	3.04 ± 0.17	735.33 ± 29.97
PBG1	3191.09	3171.14	6.13	2620
PBG2	3511.36	3466.71	6.27	2957
PBG3	3463.72	3426.47	6.34	2930
Average of PBG	3388.72 ± 172.81	3354.77 ± 160.30	6.25 ± 0.11	2835.67 ± 187.26
BG1	1577.68	1589.22	5.69	1533
BG2	1852.33	1841.75	5.46	1655
BG3	1088.24	1087.38	5.33	1074
Average of BG	1506.08 ± 387.04	1506.12 ± 383.99	5.49 ± 0.18	1420.67 ± 306.36

## Data Availability

The original contributions presented in this study are included in the article. Further inquiries can be directed to the corresponding authors.
